# Activities to support the implementation of complex interventions as part of routine care: a review of the quality of reporting in cluster randomised controlled trials

**DOI:** 10.1136/bmjopen-2015-008251

**Published:** 2015-10-09

**Authors:** Naoimh E McMahon, Emma-Joy Holland, Colette Miller, Kulsum Patel, Louise A Connell

**Affiliations:** Clinical Practice Research Unit, College of Health and Wellbeing, University of Central Lancashire, Preston, UK

## Abstract

**Objective:**

To review a sample of cluster randomised controlled trials and explore the quality of reporting of (1) enabling or support activities provided to the staff during the trial, (2) strategies used to monitor fidelity throughout the trial and (3) the extent to which the intervention being tested was delivered as planned.

**Design:**

A descriptive review.

**Data sources and study selection:**

We searched MEDLINE for trial reports published between 2008 and 2014 with combinations of the search terms ‘randomised’, ‘cluster’, ‘trial’, ‘study’, ‘intervention’ and ‘implement*’. We included trials in which healthcare professionals (HCPs) implemented the intervention being tested as part of routine practice. We excluded trials (1) conducted in non-health services settings, (2) where the intervention explicitly aimed to change the behaviours of the HCPs and (3) where the trials were ongoing or for which only trial protocols were available.

**Data collection:**

We developed a data extraction form using the Template for Intervention Description and Replication (TIDieR checklist). Review authors independently extracted data from the included trials and assessed quality of reporting for individual items.

**Results:**

We included 70 publications (45 results publications, 25 related publications). 89% of trials reported using enabling or support activities. How these activities were provided (75.6%, n=34) and how much was provided (73.3%, n=33) were the most frequently reported items. Less than 20% (n=8) of the included trials reported that competency checking occurred prior to implementation and data collection. 64% (n=29) of trials reported collecting measures of implementation. 44% (n=20) of trials reported data from these measures.

**Conclusions:**

Although enabling and support activities are reported in trials, important gaps exist when assessed using an established checklist. Better reporting of the supports provided in effectiveness trials will allow for informed decisions to be made about financial and resource implications for wide scale implementation of effective interventions.

**Strengths and limitations of this study**

- Enabling or support activities used to facilitate implementation of interventions in effectiveness trials should be described in sufficient detail to allow interpretation of results and future replication.
- In this study we used a published checklist, TIDieR (the Template for Intervention Description and Replication), to comprehensively assess the quality of reporting of enabling or support activities in cluster randomised trials.
- We did not rate the quality of reporting, as other studies have, but assessed whether or not authors reported details for items of the TIDieR checklist. The figures provided here may therefore present a more positive picture than would be the case if we assessed the extent to which interventions could be replicated from the details provided.
- Our search strategy was designed to identify a sample of effectiveness trials conducted in health services settings and a large number of eligible published trials may not have been identified potentially limiting the generalisability of the results.

## Introduction

A seminal publication in 2009 by Chalmers and Glasziou^1^ identified unusable research reports as a primary contributor to avoidable waste in research production. Despite comprehensive guidelines to assist with the reporting of clinical trials, for example the Consolidated Standards of Reporting Trials (CONSORT) 2010 statement^2^ and the Standard Protocol Items: Recommendations for Interventional Trials (SPIRIT) 2013 statement,^3^ gaps in the completeness of reports remain. Trials of complex non-pharmacological interventions have proved a particular challenge. Complex interventions are those with numerous interacting components and have been found to be adequately described in only 39% of trials.^4^

Randomised controlled trials (RCTs) have traditionally been designed to demonstrate the efficacy of complex interventions under optimum conditions.^5^ However, numerous challenges exist in attempting to translate research evidence from efficacy trials into real-life clinical practice. Most notably the lack of external validity, or generalisability, which has been a long-standing criticism of the RCT methodology.^6^ To address this issue, there has been a dramatic increase in the number of effectiveness trials being conducted which explore the degree of beneficial effect of interventions when delivered under real world conditions.^7^ RCTs are not considered to be either efficacy or effectiveness trials but can have characteristics of both and thus are said to sit on a continuum.^8^

As a result of the growing interest in more pragmatic trial designs, we have also seen an increase in the use of cluster randomisation in health services research (ie, where groups of patients, rather than individual patients, are randomised). Cluster randomised designs are particularly appropriate when there is a risk of contamination across trial groups when trial patients are managed within the same setting.^9^ Pragmatic cluster randomised trials will usually evaluate interventions when they are delivered by routinely employed healthcare professionals. Successful implementation therefore requires a change in the practices of these professionals.^10^ The ‘CONSORT’ extension for the reporting of RCTs of non-pharmacological treatments^11^ and the Template for Intervention Description and Replication (TIDieR) checklist,^12^ prompt authors to report on any activities that are used to enable or support professionals to implement the interventions being tested, and to report actual implementation of the intervention. These enabling or support activities can be considered to be interventions in themselves and therefore merit equal description in trial reports.

Previous studies exploring the completeness of intervention descriptions in clinical trials have not differentiated between different trial designs and have not explicitly assessed the completeness of reporting of enabling or support activities used to assist professionals in implementing the interventions being tested.^4^ ^13–15^ In this study we reviewed a sample of cluster RCTs, in which healthcare professionals employed to routinely deliver care were responsible for implementing the interventions being tested. We explored the quality of reporting of (1) enabling or support activities provided during the trial, (2) strategies used to monitor fidelity throughout the trial and (3) the extent to which the intervention was delivered as planned.

## Methods

### Search strategy and selection of reports of trials

To identify a sample of trial reports for the review we searched MEDLINE for publications with combinations of the search terms ‘randomised’, ‘cluster’, ‘trial’, ‘study’, ‘intervention’ and ‘implement*’ in their titles or abstracts (see online supplementary file S1). To manage the scope of the review we included reports of cluster RCTs published between 2008 and 2014. This date range was chosen as the Medical Research Council (MRC) guidance on developing and evaluating complex interventions was published in 2008,^10^ a seminal publication which has influenced the quality of reporting in trials of complex interventions. We included trials, accessible online and published in English, in which healthcare professionals (HCPs) implemented the intervention being tested as part of their routine practice. We excluded trials (1) conducted in non-health services settings (eg, schools, universities), (2) where the intervention being tested explicitly aimed to change the behaviours of the HCPs (eg, behaviour change interventions (BCIs) such as screening tools, decision aids, audit and feedback) and (3) if the trials were ongoing or for which only trial protocols were available. In instances where the intervention being tested served a dual purpose of impacting on patient outcomes, and also changing the behaviours of healthcare professionals to implement best practice, we made decisions on a case-by-case basis. Trials were included in which healthcare professionals were responsible for implementing defined actions or components of the interventions being tested. Where it was not possible to identify defined actions or components of the interventions to be implemented these trials were excluded.

NM conducted the first round of screening to remove irrelevant trials based on the exclusion criteria. In pairs, all authors independently screened the titles and abstracts of the remaining trials for inclusion. Discrepancies were resolved by discussion or by a third party. In instances where it was not possible to exclude trials based on title and abstract, full-text versions were obtained and were assessed for inclusion by the authors in the same way. Relevant publications related to the included trials (ie, trial protocols, process evaluations) were identified where possible by using the trial registration number or by manually searching for publications where the primary investigator named on the trial registration was an author.

### Data extraction and analysis

A data extraction form, based on the TIDieR checklist,^12^ was developed for the purposes of the review. All of the TIDieR items were applied to enabling and support activities. Data on fidelity was extracted at two levels (1) fidelity of implementation of the enabling and support activities and (2) fidelity of implementation of the intervention being tested. Throughout data extraction we made minor iterative changes to the way in which the items of the TIDieR checklist were organised to facilitate ease of use. For example, we reordered some of the items to fit with how they most often appeared in publications, and we separated ‘when’ and ‘how much’. Some additional information highlighted in the TIDieR checklist publication was coded as a separate item as it was reported in a number of trials (ie, competency checking is discussed under ‘who provided’ in the original TIDieR publication and we extracted this as a stand-alone item). The final data extraction form is provided in online supplementary file S2. Data were extracted on: the rationale for the support or enabling activities, materials and procedures used, where the activities were delivered, by who, when and how often and if the training was accredited or competency checked. Data on tailoring or modifications to the activities were extracted along with planned strategies to monitor implementation fidelity, and actual implementation of the intervention during the trial. Three pairs of authors independently extracted data from 15 trials each, where one author (NM) was a member of two pairs. Extracted data were cross-checked in the pairs and findings were discussed until consensus for each section was reached and the authors agreed that all potentially relevant information had been extracted.

Previous studies have rated intervention descriptions as adequate/complete or inadequate/incomplete.^4^ ^14^ Owing to the subjective nature of these types of ratings (ie, there is not yet consensus as to what information must be reported in what level of detail to constitute an adequate description of an intervention) it was decided by the study team to assess each section of the TIDieR checklist as ‘reported’ or ‘not reported’, and where information was reported to extract examples of more detailed reporting and more typical reporting. Items were assessed as ‘not reported’ if there was no mention of that item at all in the trial publications. Where authors made any reference to an item it was assessed as ‘reported’. A limitation of this approach to assessment is that it may overstate the quality of reporting in included studies. All authors independently reviewed the extracted data for each trial and made an assessment for each item extracted. Examples of variation in reporting were also highlighted by each of the review authors at this stage. Cross-checking of the assessments was carried out by the lead author. Microsoft Excel was used to analyse the data descriptively.

## Results

**Figure 1:**
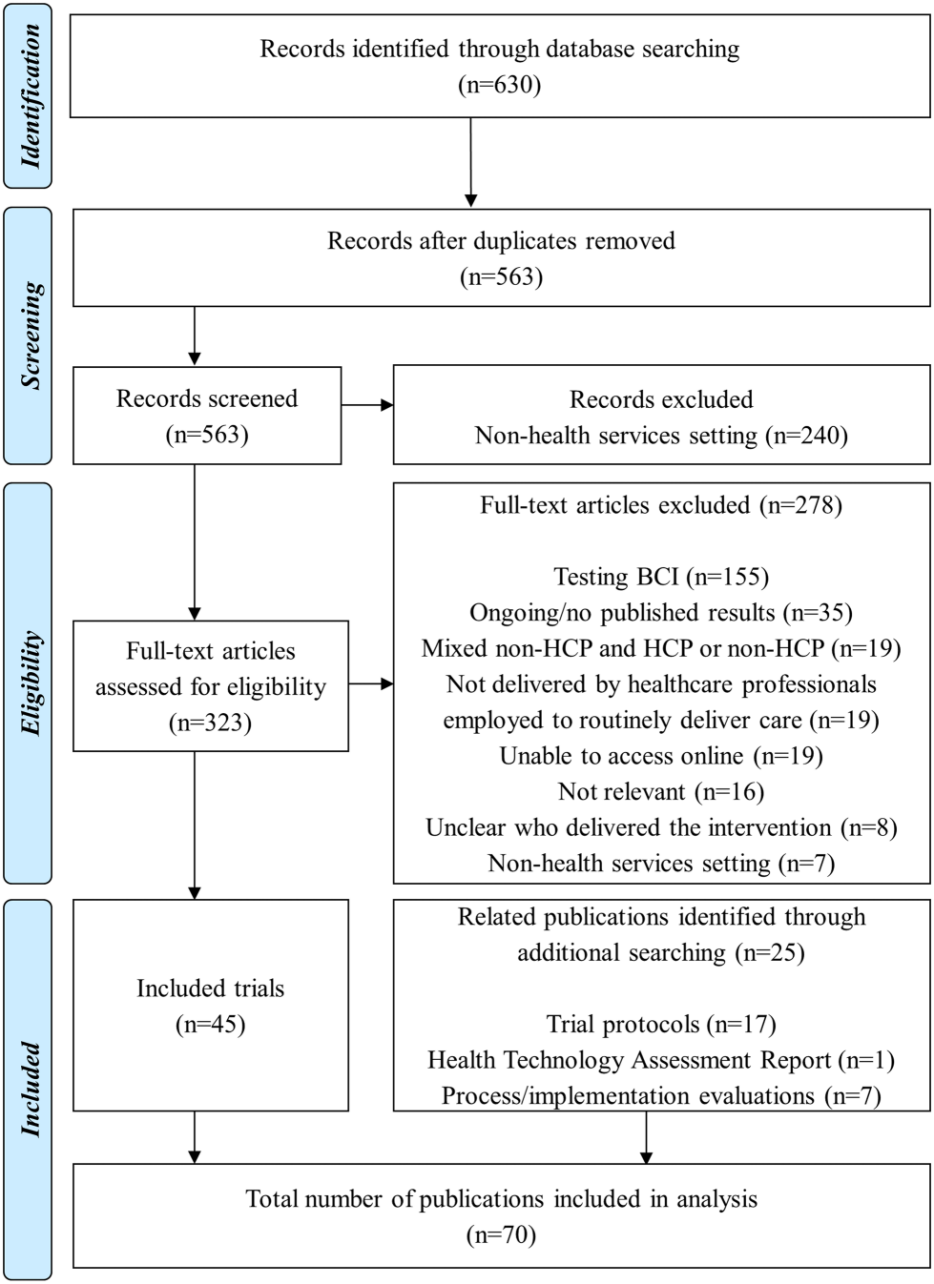
PRISMA flow diagram (BCI, behaviour change intervention; HCP, healthcare professional).

We identified 630 citations from our initial search. This became 563 when duplicate citations were removed. Trials that were conducted in a non-health service setting were excluded in the first round of screening (n=240) and the eligibility criteria applied to the remaining 323 citations. A total of 70 citations were included for data extraction (45 trial results publications and 25 related publications). Where available we used trial registration numbers to identify publications that may have been relevant for data extraction. We identified trial registration numbers for 35 of the 45 included trials. Known trial registration numbers were used to search PubMed identifying protocols and trial results publications for 26 of the included trials. The remaining publications could not be retrieved using the trial registration number alone. Identification of related publications through trial registration number search was not always successful but searching using a combination of author name, trial name and keywords identified seven additional process evaluation publications. For the 35 trials with registration numbers, 16 registrations had no records of publications, six had a results publication but no protocol, 12 had both the protocol and results publications and one was not in English. Related publications for the nine trials where registration numbers were not identified were retrieved through additional manual searching. In all but one of the related publications there was an obvious link to the original trial, that is, the trial was named or cited in the publication. One potentially related publication was not included as it was not possible to link the content to the original trial.^16^ A flow diagram of studies through the review is provided in figure 1. Citations excluded as ‘not relevant’ were secondary trial publications (eg, cost-effectiveness publications), trials where the healthcare professionals employed to deliver routine care did not take an active role in implementing an intervention (eg, system-wide quality improvement) and trials where healthcare professionals were recipients of an education or training intervention (eg, train-the-trainer initiative). All publications included in data extraction are provided in online supplementary file S3.

### Characteristics of included trials

The trials included in the review span 22 countries. The country with the most included trials was the Netherlands (n=7), followed by Australia (n=5), Spain (n=5) and the UK (n=5). The trial results publications came from 33 different journals including the *BMJ* (n=5), *PLoS One* (n=4) and the *Lancet* (n=3). The conditions most frequently studied were type 2 diabetes (n=5), asthma (n=4), cardiovascular disease, hypertension and stroke (n=4) and maternal and child health (n=4). Over half of the trials were conducted in primary care settings (n=23) and primarily evaluated lifestyle/self-management interventions (n=22). Nurses were responsible for implementing the intervention in 12 trials, general practitioners in 10 and pharmacists in 4. Further details on the characteristics of the included studies are provided in online supplementary file S4.

### Quality of reporting of enabling or support activities

Forty of the 45 trials (88.8%) made reference to some form of enabling or support activities conducted during the trial. Of the five trials that did not describe any enabling or support activities, three of these were evaluating infection control interventions in hospital settings. The number of trials providing information on each of the items of the TIDieR checklist, adapted for enabling or support activities, is shown in table 1. How activities were provided, and how much, were the most frequently reported items (75.6% and 73.3% of trials, respectively). Reporting of the rationale for the chosen activities, any tailoring or modifications of the activities and measures of planned and actual activities carried out during the trial (eg, attendance at training sessions) were the least frequently reported.

**Table 1:** Number of trials reporting components of enabling or support activities

Components of enabling or support activities	Reported	
	Per cent	n
Why	11.1	5
Who provided	53.3	24
What materials	57.8	26
Where	22.2	10
When	51.1	23
How	75.6	34
How much	73.3	33
Tailoring	4.4	2
Modifications	4.4	2
Measure of fidelity	2.2	1
Actual activities delivered	6.7	3
Competency checking	17.8	8

### Quality of reporting of strategies used to monitor fidelity throughout the trial and the extent to which the intervention was delivered as planned

Over half of the included trials (n=29) reported monitoring the extent to which healthcare professionals delivered the intervention as planned within the trial. The ways in which fidelity was most frequently monitored included notes audits (n=12), rating of random samples of audio-recorded sessions (n=5), self-reported measures of implementation (from healthcare professionals and study participants) (n=4) and observation of practice (n=2). Of the trials that reported collecting measures of fidelity, data from these measures was reported in 44.4% (n=20) of the trials. Examples of reporting, and variation across trials for the individual items are provided in online supplementary file S5.

## Discussion

### Statement of principal findings

Almost all of the trials (89%) included in this review reported that enabling or support activities were used to facilitate implementation of the intervention being tested. However, large discrepancies were identified in the quality of reporting of the components of these activities. Authors most frequently reported how enabling or support activities were provided, and how much was provided, but details related to who provided, where, when and the underpinning rationale for the content and delivery of the activities were often omitted. Less than 20% of the included trials reported that competency checking took place prior to implementation and data collection. Although, 29 trials reported using specific fidelity measures to assess the extent to which the healthcare professionals had implemented the intervention as planned during the trial, data from these measures was not reported in nine instances.

### Strengths and weaknesses of the study

A strength of this study is the use of the published TIDieR checklist (the Template for Intervention Description and Replication) to assess the quality of reporting of the included trials.^12^ TIDieR has been developed by Hoffmann and colleagues as an extension to Item 5 of the CONSORT Statement and aims to guide authors in describing interventions in a level of detail to allow for future replication. Although the primary purpose of the TIDieR checklist is to assist in the describing of interventions being tested, a number of items in TIDieR discuss how some interventions, particularly complex ones, might require additional activities to enable or support the intervention to occur, and these should also be described. We would argue, and have demonstrated here, that these activities are *interventions* in themselves and therefore can be reported with the same level of detail and attention afforded to the intervention under investigation.

A limitation of this review may be the included trials and the way in which they were identified. Although a systematic process was used to identify effectiveness cluster RCTs, a large number of published trials will not have been identified by our search (eg, due to the time period or search terms used). The results of our review therefore may not be generalisable. However, it is also unknown whether a larger sample would have influenced the overall results of the study. When attempting to identify secondary publications for the included trials it became evident that authors do not always include the trial registration numbers in the trial results publications. We carried out additional searching using publication titles, trial names and author names but were unable to locate trial registration numbers for 10 of the included trials. It is not possible to conclude whether these trials were not registered or whether the search efforts were unable to locate registration details. We endeavoured to retrieve all relevant publications, including process evaluations and reports on training procedures. However, it is possible that details of support activities may be reported in publications not identified during our searches. It was interesting to note that in a number of instances process evaluation publications did not provide additional information on actual implementation during the trial or associated measures. The focus of these publications was often on providing in depth qualitative accounts on stakeholders experiences of the implementation process. MRC guidance on process evaluations was published earlier this year and may result in greater consistency as to the type of information reported in these evaluations.^17^

As previously mentioned, similar trials^4^ ^13^ ^14^ have not only looked at whether or not details have been reported but have also made a judgement as to whether the details provided were sufficient to merit replication. In this review we opted to assess whether or not authors provided details on the items of the TIDieR checklist and provide supporting examples. This may also be a limitation of the review as the figures provided here without doubt present a more positive picture than would be the case if we were to make subjective judgements on the extent to which interventions could be replicated from the details provided.

### Meaning of the study

Despite improvements in the quality of reporting of clinical research in recent years, complex trial designs and interventions have resulted in trial reports leaving readers with many questions unanswered. In this review we have focused in particular on the reporting of enabling and support activities and implementation fidelity within trials, as this is important information for both interpreting the research findings and also for clinicians wanting guidance on how to implement interventions in their practice. We have shown that although these activities are reported in trials, there exist important gaps when assessed using items of the TIDieR checklist. Implementation science is a growing field of research, with much interest in how we can best translate interventions that have been shown to be effective in research into day-to-day clinical practice. In order to progress this field of research it is important that authors describe the rationale for their implementation activities, and the components of these activities in order to contribute to the growing evidence base. Publication restrictions pose a particular challenge in achieving this goal and therefore novel methods of efficiently communicating these details in useable formats are urgently needed.

### Unanswered questions and future research

Effectiveness cluster randomised trials of complex non-pharmacological interventions pose numerous challenges in their design, implementation and reporting. However, there is a growing appreciation for the nuanced and iterative processes that occur when introducing a new intervention into routine clinical practice and the role of contextual and personal factors.^18^ There is a need for more transparent reporting about what is happening in trials to make informed decisions about the feasibility and effectiveness of interventions, along with their likely financial and resource implications. There is also a need to establish consensus about the level of detail that should be reported for such large scale trials to ensure that what is reported is meaningful and useful for patients, the public, clinicians and researchers.
